# Bibliometric analysis and knowledge mapping of diabetes mellitus combined with tuberculosis research: trends from 1995 to 2023

**DOI:** 10.3389/fimmu.2025.1571123

**Published:** 2025-04-04

**Authors:** Yufeng Li, Ruizi Ni, Yajing An, Ling Yang, Zhaoyang Ye, Li Zhuang, Linsheng Li, Liang Wang, Wenping Gong

**Affiliations:** ^1^ Institute of Tuberculosis, Senior Department of Tuberculosis, the Eighth Medical Center of PLA General Hospital, Beijing, China; ^2^ Graduate school, Hebei North University, Zhangjiakou, Hebei, China; ^3^ Department of Geriatrics, the Eighth Medical Center of PLA General Hospital, Beijing, China

**Keywords:** bibliometrics, diabetes mellitus, tuberculosis, diabetes mellitus complicated with tuberculosis (DM-TB), research trends

## Abstract

**Background:**

The synergistic epidemic of diabetes mellitus and tuberculosis (DM-TB) has created a dual disease burden, challenging global health systems with complex pathophysiological interactions and suboptimal treatment outcomes. To decode the evolving research landscape, this study presents the latest comprehensive bibliometric analysis mapping the intellectual architecture of DM-TB research over three decades.

**Methods:**

We systematically analyzed 791 peer-reviewed articles from the Web of Science Core Collection (1995-2023) using CiteSpace, VOSviewer, and Bibliometrix. Advanced metrics including co-citation networks, keyword burst detection, and institutional collaboration patterns were employed to identify paradigm-shifting trends.

**Results:**

Three distinct growth phases were observed: initial stagnation (1995-2007, <10 annual publications), exponential growth (2008-2019), and research diversification (2020-2023). The United States dominated scientific output (27.3% of total publications), while the London School of Hygiene & Tropical Medicine emerged as the central hub for international collaborations (TLS=176). Keyword evolution revealed three transformative phases: (1) Pathomechanistic exploration (1995-2016): Focused on hyperglycemia-immunity interplay and epidemiological surveillance; (2) Translational innovation (2017-2020): Shifted to preclinical models, pharmacokinetic optimization, and multidrug resistance; (3) Precision medicine era (2021-2023): Emerging hotspots in latent TB screening (burst strength=6.82), metformin-mediated immunomodulation, and AI-driven diagnostic biomarkers.

**Conclusion:**

Beyond delineating historical trajectories, this study identifies critical knowledge gaps in inflammation-resolution mechanisms and insulin resistance pathways, proposing a roadmap for targeted biomarker discovery and global health policy formulation. The constructed knowledge framework empowers strategic resource allocation for combating the DM-TB syndemic.

## 1 Introduction

Diabetes mellitus (DM) is a group of metabolic disorders characterized by persistent hyperglycemia due to defects in insulin secretion or impaired insulin action. This chronic hyperglycemic state is closely associated with long-term damage, dysfunction, and failure of various organs, posing a significant threat to human health (1). According to estimates by the International Diabetes Federation (IDF), in 2021, the global number of people with diabetes reached 537 million, with diabetes-related health expenditures amounting to USD 966 billion, and this figure is projected to exceed USD 1,054 billion by 2045, highlighting the immense economic burden of diabetes on global healthcare systems (2). The Global Burden of Disease (GBD) report indicates that the prevalence of DM is rising at an alarming rate, with an estimated over 1.31 billion people expected to be affected by 2050 (3). Hyperglycemia negatively impacts immune responses, making diabetic patients more susceptible to infections (4).

Tuberculosis (TB) is a pulmonary infectious disease caused by *Mycobacterium tuberculosis* (MTB) (5). The World Health Organization (WHO) Global Tuberculosis Report 2024 reveals that in 2023, there were an estimated 10.8 million new TB cases globally, with 1.25 million deaths, underscoring TB as a major public health challenge worldwide (6, 7). Existing research indicates a close association between DM and TB (8), TB can lead to abnormal glucose metabolism, becoming a significant factor in poor glycemic control, while hyperglycemia weakens immune defenses, promoting the growth and proliferation of MTB, thereby creating a vicious cycle (9, 10). A meta-analysis indicates that the global prevalence of diabetes mellitus complicated with tuberculosis (DM-TB) co-occurrence reaches 13.73% (11). The increase in DM-TB poses a serious threat to human life and health and imposes a significant economic burden on global healthcare systems.

Despite extensive research focusing on DM-TB, particularly in terms of its epidemiology, treatment management, and pathogenesis (12–14), the relationship between DM and TB remains incompletely understood. Bibliometric analysis is a method involving the statistical analysis of research literature related to a specific domain or topic, providing insights into the current state of research and its temporal trends, thus offering researchers macro-level information (15). It helps researchers understand progress trends and key areas of research, clarify research directions, and explore new research foci (16). This study employs bibliometric methods to analyze the overall status of DM-TB research, examining current hotspots and trends in DM-TB research.

## 2 Materials and methods

### 2.1 Data sources and retrieval strategy

**Figure 1:**
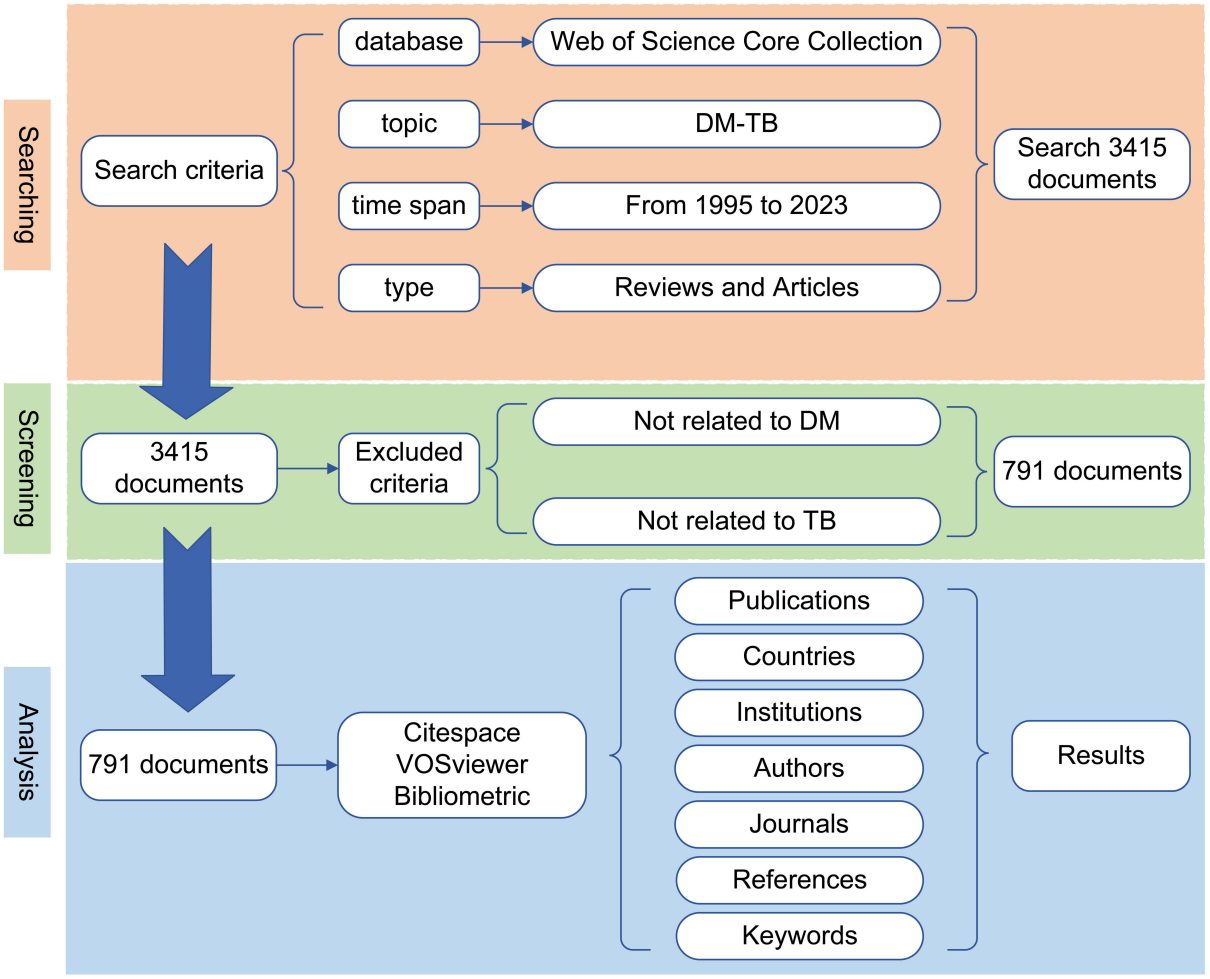
Search strategy and flowchart. Employing the aforementioned search strategy in the Web of Science Core Collection (SCI-Expanded) database, a total of 791 publications were retrieved and subsequently exported to VOSviewer, CiteSpace, and Bibliometrix for bibliometric analysis.

This study conducted data retrieval from the Web of Science Core Collection (WoSCC) database, which provides the document formats required for bibliometric analysis software such as CiteSpace, VOSviewer, and Bibliometrix R package, making it one of the most widely used databases in bibliometric studies (17). We selected the Science Citation Index Expanded (SCI-E) from the WoSCC database as the primary source of literature. Since only publicly available data from the WoSCC database was used in this study, there are no ethical approval issues. Detailed retrieval strategies are provided in the **Supplementary Material Box 1**. The document types were restricted to Articles and Reviews, with the time span set from January 1, 1995, to December 31, 2023. As of September 23, 2024, an independent literature search was conducted, identifying a total of 791 related papers, and all retrieved documents were exported and saved in TXT format for subsequent bibliometric analysis. The complete retrieval process is detailed in **Figure 1**.

### 2.2 Inclusion and exclusion criteria

Inclusion criteria: (1) Papers indexed in the SCI-E database between 1995 and 2023; (2) Research papers related to DM-TB. Exclusion criteria: (1) Papers unrelated to DM-TB; (2) Non-scholarly articles such as conference papers, news reports, book chapters and so on. (3) Duplicate publications, where the same research results are reported in different journals or conferences.

### 2.3 Data visualization and analysis

The downloaded data was imported into Microsoft Office Excel 2021, CiteSpace, VOSviewer, Bibliometrix and OriginPro 2024 for analysis to extract meaningful information. Specifically, Microsoft Office Excel 2021 was employed for publication trend statistics, data collation and related tables. VOSviewer (version 1.6.20) was used to construct visualization maps for collaboration networks, co-citation analysis, and keyword co-occurrence analysis among countries/regions, institutions, journals, and cited journals (18). CiteSpace (version 6.3.R1) was employed for journal dual-map overlay analysis, centrality of countries, citation clustering and burst analysis, and keyword clustering and burst analysis (19). Additionally, R studio desktop software (2024.04.2) was used to connect R software Bibliometrix (version 4.4.1) for visual analysis of annual publications, author analysis, journal analysis country distribution and collaboration networks, and thematic evolution analysis (20). OriginPro 2024 was used to construct lollipop charts of author publication output.

## 3 Results

### 3.1 Annual publication output analysis

**Figure 2:**
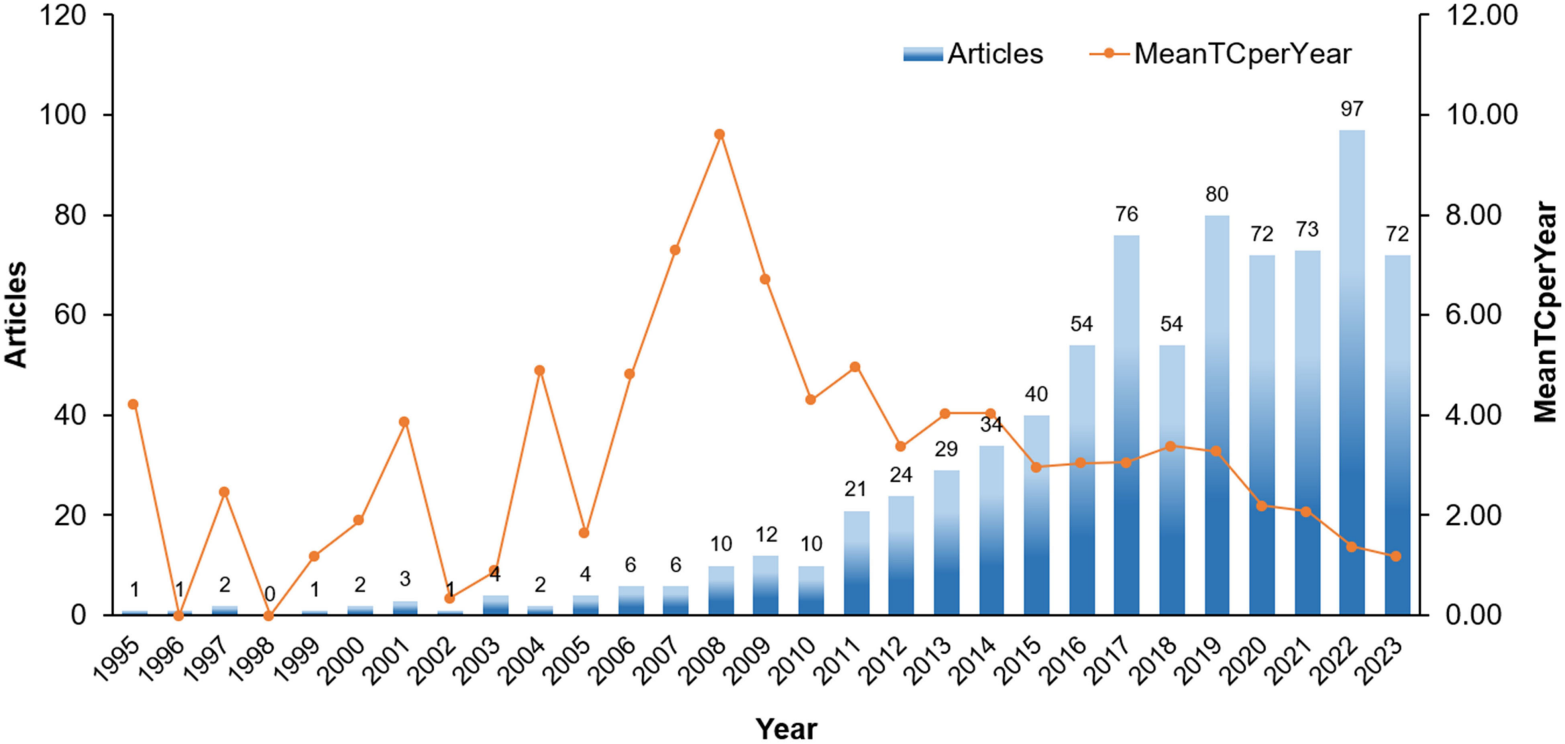
Growth trend of publications. Temporal trends in DM-TB research publication growth (1995-2023): Annual publication volume and mean total citations per year.

From 1995 to 2023, a total of 791 papers were published in the field of DM-TB, comprising 702 original articles and 89 reviews. These publications involved 4,062 authors across 1,430 institutions from 100 different countries/regions. **Figure 2** illustrates the annual publication volume of DM-TB related articles. The number of publications generally showed an upward trend from 1995 to 2023, despite minor fluctuations. Three distinct phases were identified: Phase I (1995-2007), Phase II (2008-2019), and Phase III (2020-2023). Phase I maintained annual publications <10, representing the initial stage of DM-TB research; Phase II demonstrated substantial growth with an average of 37 publications per year; Phase III sustained steady growth in annual output, peaking at 97 publications in 2022. The Mean Total Citation per Year (MeanTCperYear) was used to evaluate the impact of publications. MeanTCperYear is defined as the total citation count of a publication divided by the cumulative years since its publication year. From 1995 to 2006, the MeanTCperYear remained below 5, indicating relatively stable impact. In 2008, the index peaked at 9.61, showing a significant increase in publication impact at that time. However, a declining trend was observed subsequently, with the MeanTCperYear dropping to 1.18 by 2023, reflecting a need for more impactful and high-quality research in this field.

### 3.2 Country or region analysis

**Figure 3:**
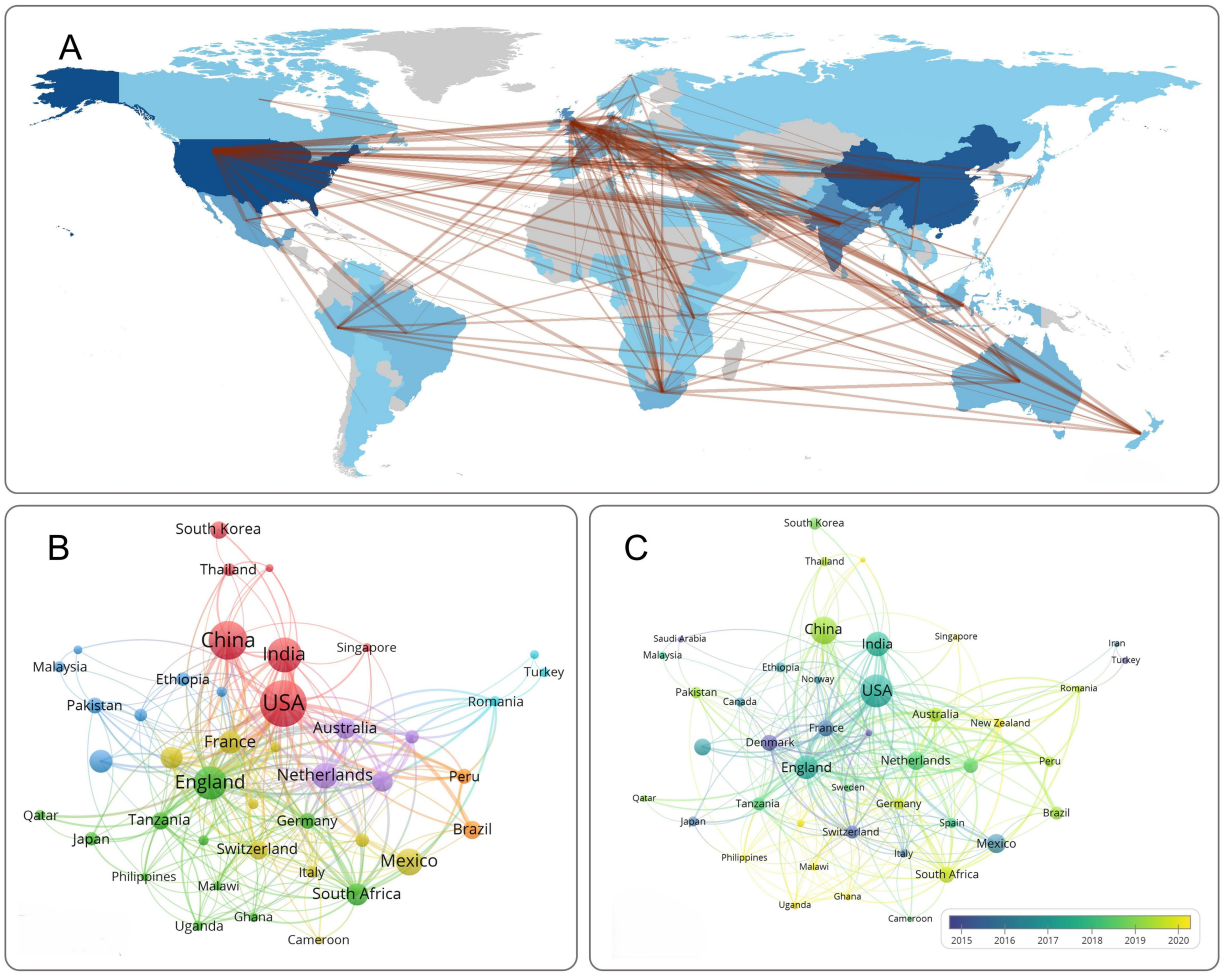
Visualization of countries/regions analysis. **(A)** Country collaboration map. The colors of the map’s regions indicate the number of published articles, with darker shades corresponding to a higher volume of articles contributed by each country/region. **(B)** Network visualization of countries. **(C)** Overlay visualization of countries. Nodes represent countries/regions, with node size corresponding to publication count. Connections between nodes indicate collaborative partnership.

A total of 100 countries/regions have conducted research on DM-TB. **Table 1** lists the top 10 countries/regions in terms of publication volume, with the United States leading (216 articles), followed by China (145 articles), India (116 articles), the United Kingdom (103 articles), and Mexico (66 articles). Betweenness centrality (BC) quantifies a node’s frequency of occurring on shortest network paths, assessing its topological significance, with values exceeding 0.1 designating pivotal nodes (21). Five countries had BC values greater than 0.1: the United States (0.33), the United Kingdom (0.29), South Africa (0.14), Denmark (0.12), and France (0.11), indicating their crucial role in research in this field. The United States led in publication volume (216 articles), BC (0.33), and citation count (8,970), significantly surpassing other countries/regions. Although China and India ranked highly, their BC was relatively low. **Figure 3A** maps out the collaborative relationships between major countries/regions. The width of the connecting lines represents the strength of collaboration, with wider lines indicating stronger partnerships. The United States is the most frequent initiator and participant in collaborations, particularly with India, the United Kingdom, China, Mexico, Brazil, Peru, South Africa, and Australia. **Figure 3B** illustrates the collaboration network for countries/regions with a minimum of five publications. Additionally, the node colors in **Figure 3C** represent the year each country first appeared in this research field, with yellow nodes indicating more recent entry. Since 2020, some countries/regions have entered this research area, showing increasing global interest in the field.

**Table 1:** Ten countries with the most publications.

Rank	Country	Documents ^a^	Betweenness Centrality ^b^	Citations ^c^	TLS ^d^
1	USA	216	0.33	8970	266
2	China	145	0.02	1985	94
3	India	116	0.05	2621	117
4	England	103	0.29	3905	280
5	Mexico	66	0.06	1844	33
6	Netherlands	58	0.04	1963	156
7	France	47	0.11	2478	131
8	South Africa	44	0.14	741	110
9	Denmark	43	0.12	2328	101
10	Indonesia	37	0.03	1309	100

a Documents: The number of publications produced by each country.

b Betweenness Centrality: This metric describes the degree or importance of a node’s position within a network. Nodes with higher betweenness centrality are typically in more significant positions, exerting greater influence or control within the network.

c Citations: The number of times publications from each country have been cited.

d TLS (Total Link Strength): TLS is a metric that measures the strength of connections in a network, here referring to the strength of connections between countries.

### 3.3 Institutional and collaborative institution analysis

A total of 1,430 institutions conducted research on DM-TB. **Table 2** lists the top ten institutions by publication volume, with the London School of Hygiene & Tropical Medicine (52 articles), International Union Against TB and Lung Disease (33 articles), and University of Massachusetts (32 articles) ranking as the top three. Analyzing collaboration intensity, the top three institutions in total link strength (TLS) were London School of Hygiene & Tropical Medicine (176), International Union Against TB and Lung Disease (111), and St George’s, University of London (108). Notably, the London School of Hygiene & Tropical Medicine stood out with significantly higher citation frequency and TLS than the other two, indicating its leading impact in publications.

**Table 2:** Ten institutions with the most publications.

Rank	Institution	Country	Documents	Citations	TLS
1	London School of Hygiene & Tropical Medicine	England	52	2264	176
2	International Union Against TB and Lung Disease	France	33	2094	111
3	University of Massachusetts	USA	32	1253	99
4	Radboud University Nijmegen	The Netherlands	30	1328	103
5	National Institute for Research in Tuberculosis	India	28	828	73
6	St George’s, University of London	England	26	644	108
7	National Taiwan University	China	21	764	43
8	NIH National Institute of Allergy And Infectious Diseases(NIAID)	USA	21	532	51
9	Prof. M. Viswanathan Diabetes Research Centre	India	21	464	67
10	Emory University	USA	20	380	24
11	University of Copenhagen	Denmark	20	503	69
12	World Diabetes Foundation	Denmark	20	1770	82

**Figure 4:**
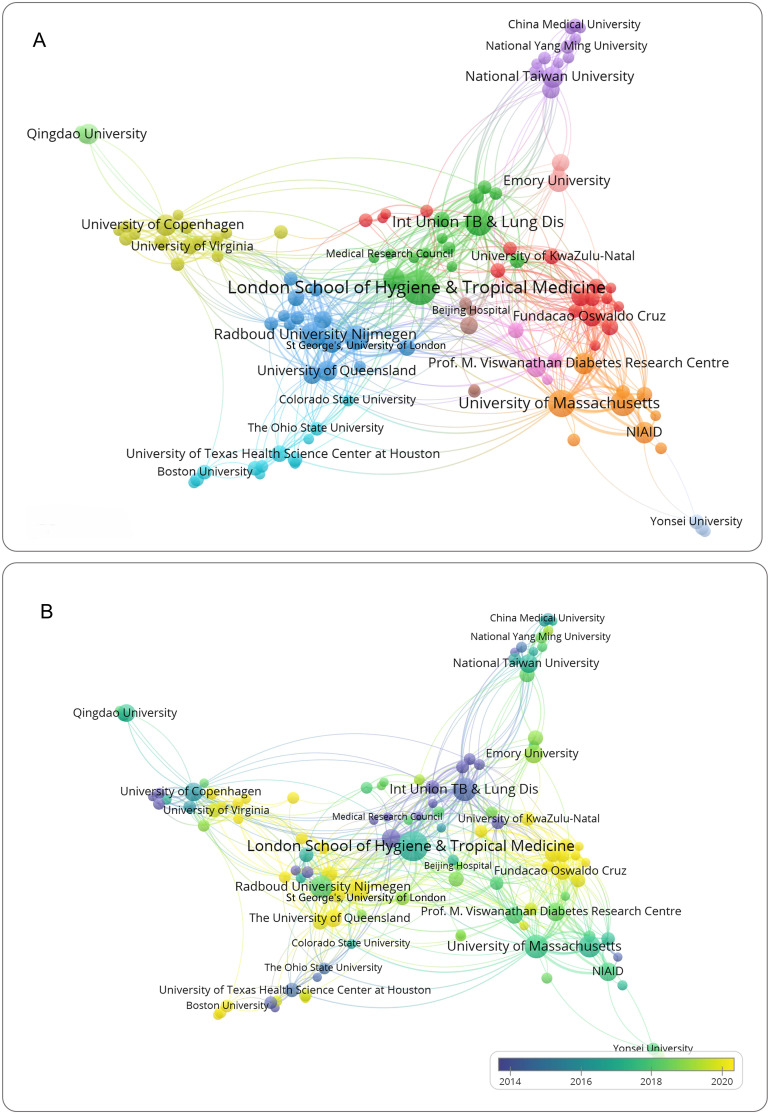
Visualization of institutions analysis. **(A)** Network visualization of institutions. **(B)** Overlay visualization of institutions. Nodes represent institutions, with node size corresponding to publication count. Connections between nodes indicate collaborative partnership.

Co-occurrence analysis on institutions with more than five publications is shown in **Figure 4A**, where apparent close collaborations exist among these institutions. Institutions like London School of Hygiene & Tropical Medicine, Radboud University Nijmegen, and University of Copenhagen have gradually formed self-centered collaborative networks. In **Figure 4B**, nodes predominantly purple, such as International Union Against TB and Lung Disease, indicate early entry into the field, while nodes primarily yellow, like University of Queensland, Fundação Oswaldo Cruz, and University of Virginia, indicate later entry. The largest yellow node, St George’s, University of London, suggests it might be an emerging research institution in the field, potentially leading future developments and becoming a key force.

### 3.4 Author analysis

**Figure 5:**
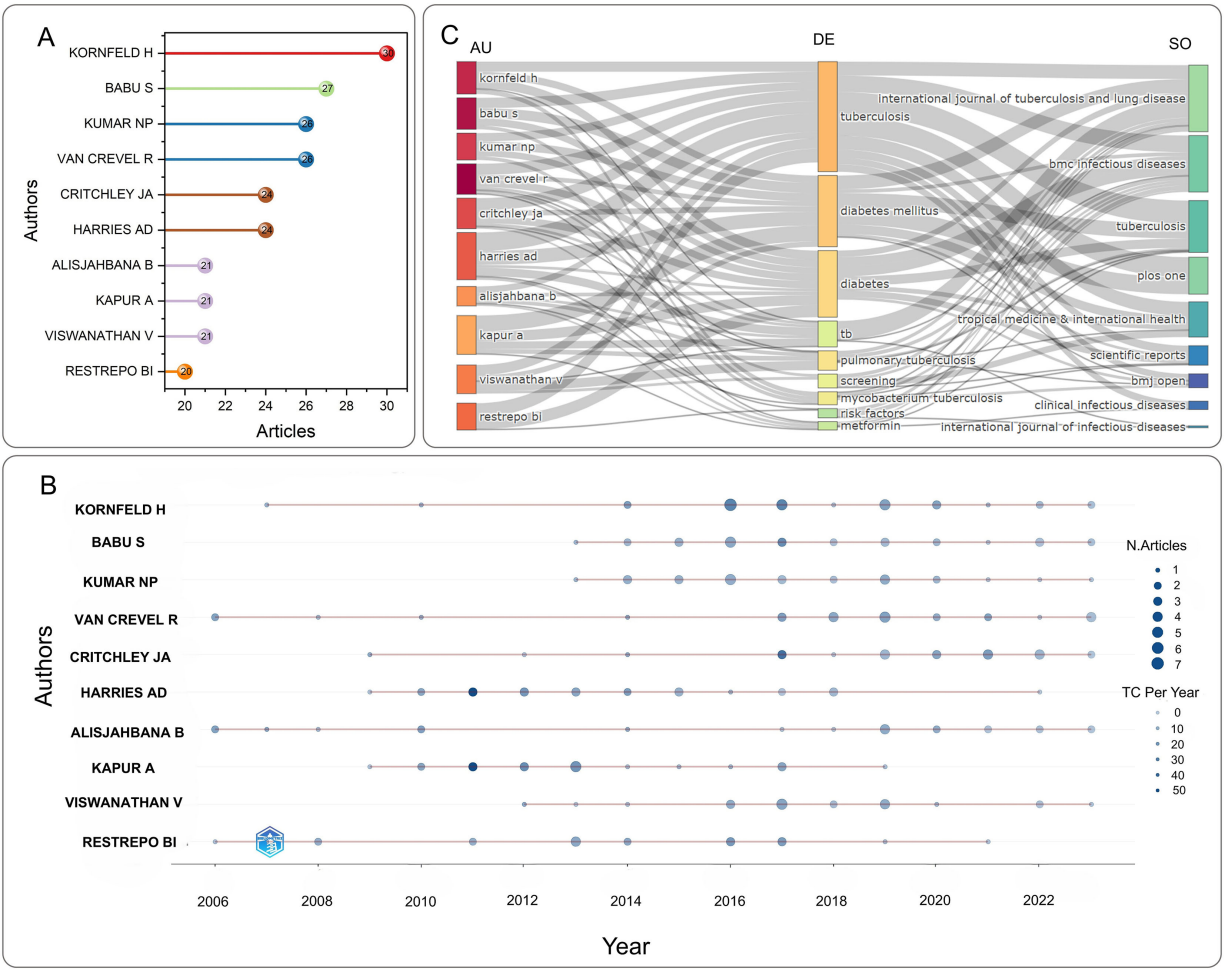
Visualization of authors analysis. **(A)** Top 10 most relevant authors in the field of DM-TB. **(B)** Authors’ production over time. Node size corresponds to publication count, and color denotes annual total citation counts per author **(C)** Three-field plot illustrating interconnections among AU (Author), DE (Keyword), and SO (Journal).

A total of 4,062 authors have conducted research on DM-TB. **Supplementary Table S1** and **Figure 5A** provide information about the top ten authors with the most publications. The H-index, G-index and M-index are comprehensive quantitative indicators considering both the quantity and quality of academic output, used to assess academic impact (22–24). Among them, Kornfeld, Hardy has the highest publication count (30 articles), higher H-index (18), and highest G-index (30). In contrast, Kapur, Anil achieves the highest H-index (20) and total citation count (1,829), with a marginally higher M-index (1.25 vs. 1.0). These metrics collectively establish that Kornfeld, Hardy represents the most prolific author with dominant core paper impact in DM-TB research, while Kapur, Anil demonstrates the highest comprehensive academic influence. This discrepancy may be attributed to Kapur’s institutional leadership as Chair of the World Diabetes Foundation, which enhances academic visibility through frequent conference participation, collaborative networks, and sustained research engagement. **Figure 5B** compares authors’ publication output over time, with Kornfeld, Hardy having the most papers, and Van Crevel, Reinout, Restrepo, Blanca I and Alisjahbana Bachti starting research earliest and having the longest duration. Recently, Critchley, Julia A and Van Crevel, Reinout have made greater contributions. **Figure 5C** uses a three-field plot to illustrate authors’ publishing patterns in different related topics and journals. The top 10 authors’ main research keywords include “tuberculosis”, “diabetes mellitus”, “pulmonary tuberculosis”, “tb”, “screening”, “mycobacterium tuberculosis”, “metformin”, “risk factors”. Their work is typically published in journals like the International Journal of Tuberculosis and Lung Disease, BMC Infectious Diseases, Tuberculosis and Plos One.

### 3.5 Journal and cited journal analysis

**Figure 6:**
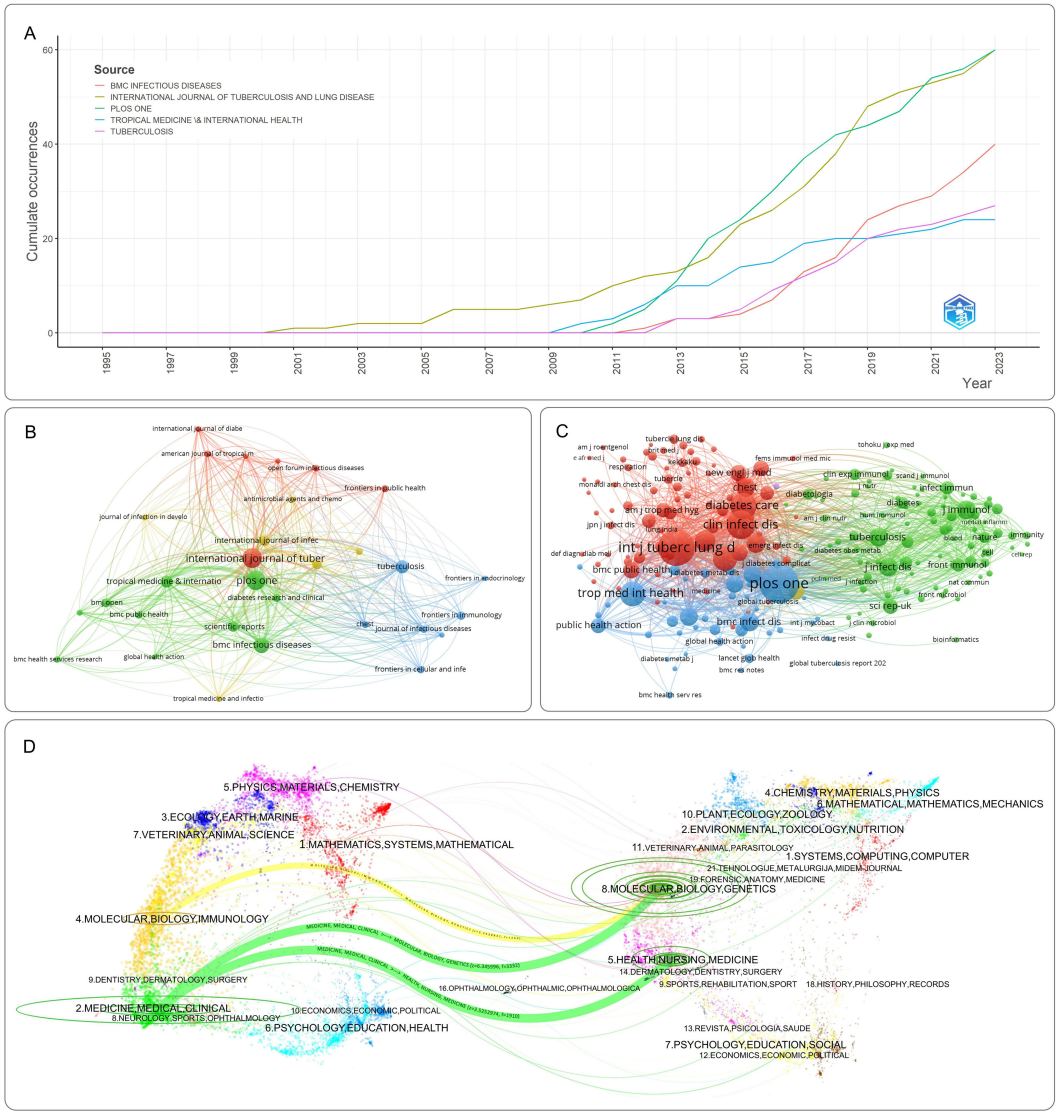
Visualization of journal analysis. **(A)** Top 10 journals’ production over time. **(B)** Network visualization of journals. **(C)** Network visualization of co-cited journals. Nodes represent journals or co-cited journals, with node size corresponding to publication count. Connections between nodes indicate collaborative partnership. **(D)** The dual-map overlay of journal publishing research. The citing journals mainly focus on molecular, biology, immunology, medicine, medical and clinical studies; the cited journals primarily focus on health, nursing, medicine, molecular biology, and genetics.

The 791 publications related to DM-TB spanned 263 journals and 4,790 co-cited journals. **Supplementary Tables S2** and **S3** list the top ten journals by publication volume and citation frequency, respectively. The journals with the most publications are International Journal of Tuberculosis and Lung Disease (60 articles) and PLOS ONE (60 articles), followed by BMC Infectious Diseases (39 articles). The highest impact factor (IF) among them belongs to Clinical Infectious Diseases at 8.2. A journal’s impact largely depends on its citation frequency, as citations reflect the extent to which scholars and researchers in the field refer to its articles. PLOS ONE has the highest citation frequency (1574 times), followed by International Journal of Tuberculosis and Lung Disease (1,379 times) and Clinical Infectious Diseases (824 times), with six journals in the Q1 category, indicating significant impact in the field. **Figure 6A** shows cumulative publications from the top five journals. Before 2013, the International Journal of Tuberculosis and Lung Disease led in publications; after 2013, publication volumes for various journals increased markedly, with International Journal of Tuberculosis and Lung Disease and PLOS ONE taking the lead.

Cluster analysis of journals and co-cited journals is presented in **Figures 6B, C**, visually displaying collaboration among journals and co-cited journals. Dual-map overlay of journals illustrates the relationship between citing and cited journals, with research areas of citing journals on the left and cited journals on the right. As shown in **Figure 6D**, the map identifies three main citation paths, including one orange path and two green paths. The orange path indicates that journals from the fields of molecular, biology and genetics are most likely to be cited by journals in the fields of molecular, biology and immunology. The green path suggests that publications related to molecular, biology and genetics, as well as health, nursing, and medicine fields are more likely to be cited by journals in the fields of medicine, medical and clinical.

### 3.6 Co-cited references analysis

**Supplementary Table S4** lists the top ten most cited articles among the 791 publications. These articles systematically investigated the bidirectional relationship between DM-TB, including DM’s elevated risk of developing TB, its impacts on therapeutic responses and clinical outcomes, as well as their epidemiological interactions and clinical management strategies (25–34). “Diabetes mellitus increases the risk of active tuberculosis: a systematic review of 13 observational studies” is the most cited article (416 times). This paper systematically reviews and meta-analyzes observational studies evaluating the association between DM and TB, finding that DM is associated with increased TB risk, and indicating that TB control should consider interventions targeting DM patients (25). The second most cited article (272 times), “The impact of diabetes on tuberculosis treatment outcomes: a systematic review” systematically reviews 33 observational studies on DM patients receiving TB treatment, finding that DM increases the risk of treatment failure, death, and relapse in TB patients. This study highlights the need for increased attention to the treatment of TB in diabetic patients, potentially including testing for suspected DM, improving glycemic control, and enhancing clinical and treatment monitoring (26). “Tuberculosis and diabetes mellitus: convergence of two epidemics” is the third most cited article (211 times). It reviews the epidemiology of TB and DM, and outlines the role of DM in TB susceptibility, clinical manifestation, and treatment response. Additionally, it reviews the potential mechanisms by which DM leads to TB, the impact of TB on DM control, and pharmacokinetic issues related to the co-management of DM and TB (27).

**Figure 7:**
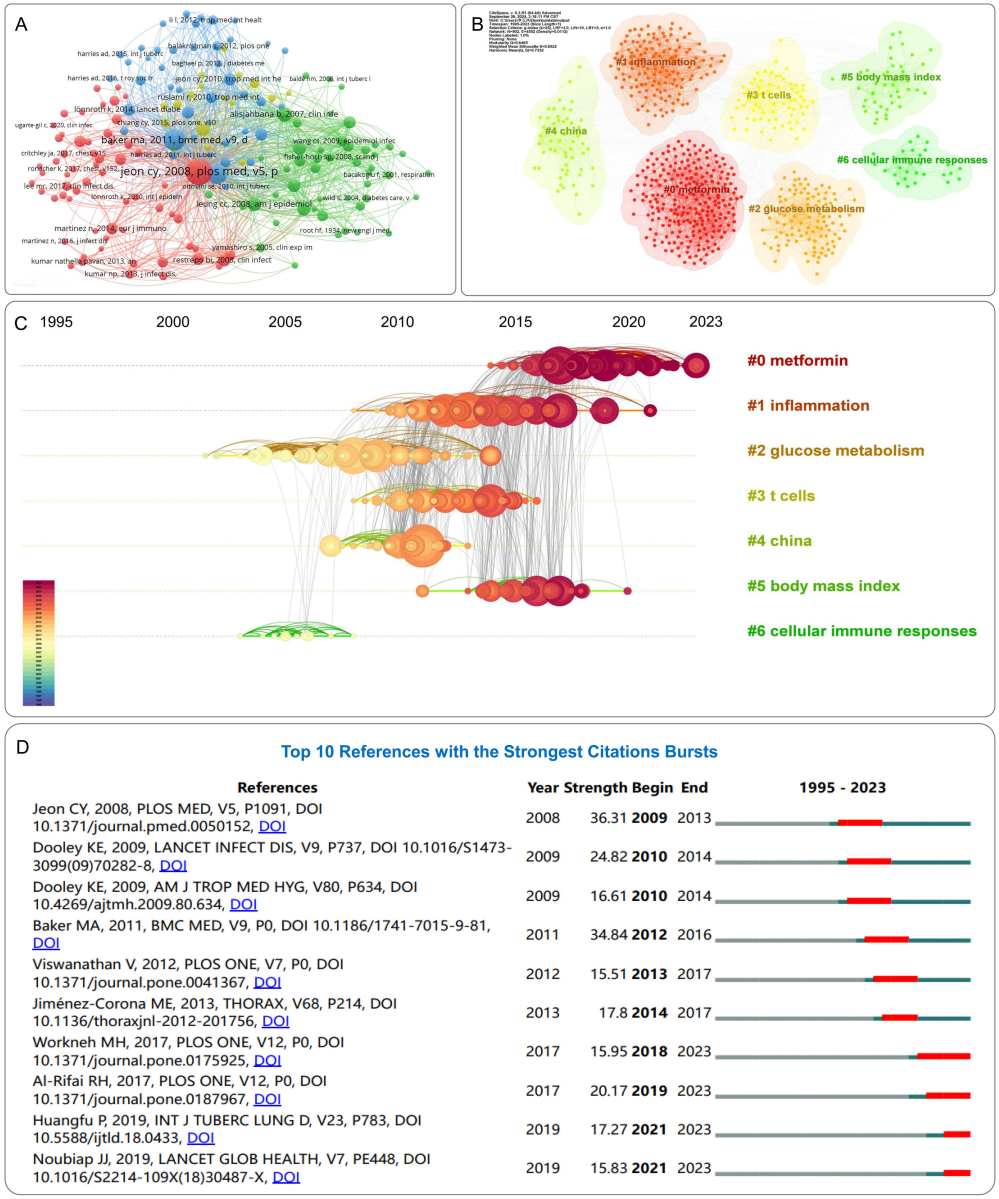
Visualization of reference analysis. **(A)** Network visualization of references. **(B)** Clusters of references. **(C)** Reference timeline view. Nodes represent cited references, where the diameter proportionally correlates with citation frequency. A chromatic temporal gradient from blue (1995) to red (2023) visually encodes the publication timeline across nodes. **(D)** Top 10 references with the strongest citation bursts. Red bars represent the duration of burst periods. The burst strength demonstrates the significance of this study to the research field.

Co-citation analysis of cited articles using VOSviewer is shown in **Figure 7A**, with the minimum citation threshold set to 20. Network clustering of literature relationships using CiteSpace is depicted in **Figure 7B**, with the first seven clusters shown. The parameters were configured as follows: temporal scope spanning 1995–2023 with 1-year slices, node type defined as references, g-index standardization (k=25), and pruning disabled. The Modularity Q value of the clustering map is 0.6465, and the Weighted Mean Silhouette S value is 0.8522, confirming the reliability of the clustering structure. Cluster #0: “metformin” is the largest cluster, indicating its extensive citation. Followed by clusters such as “inflammation” (#1), “glucose metabolism” (#2), “T cells” (#3), “China” (#4), and “body mass index” (#5). Other important clusters include “cellular immune responses” (#6). The cluster timeline diagram in **Figure 7C** illustrates the evolution of research directions, with nodes representing cited articles, different shades representing research evolution, and curves between nodes indicating co-citation relationships. Reference burst analysis enables quick identification of the most influential cited documents, facilitating a clear understanding of the DM-TB research backdrop and current frontlines and emerging trends. Reference burst analysis is shown in **Figure 7D**, where higher burst strength values indicate explosive increases in citations during specific periods. Jeon CY et al.’s study (25) shows the highest burst strength, confirming the association between DM and increased TB risk, significantly impacting the DM-TB field. Recent studies by Workneh MH (35), AI-Rifai RH (36), Huangfu P (37), and Noubiap JJ (38) are the latest, exploring the prevalence and mortality of DM-TB.

### 3.7 Keywords analysis

**Figure 8:**
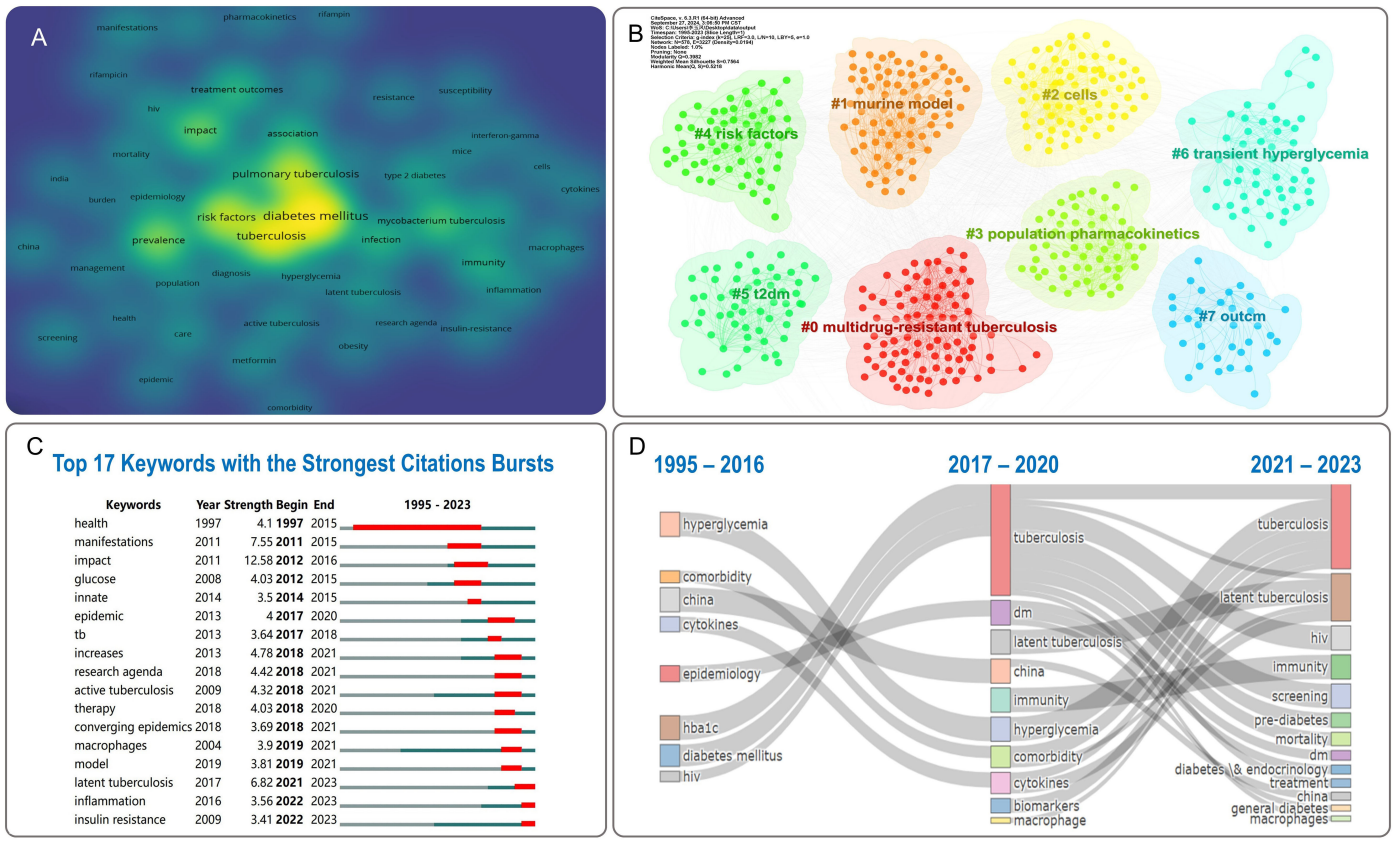
Visualization of keywords analysis. **(A)** Density visualization of keywords. **(B)** Clusters of keywords. **(C)** Top 17 keywords with the strongest citation bursts. Red bars represent the duration of burst periods. The burst strength demonstrates the significance of this study to the research field. **(D)** Thematic evolution of DM-TB: temporal dynamics of Keywords.

To understand the core content and hotspots in the DM-TB field, we analyzed author keywords in the retrieved literature. We extracted 2,030 keywords from 791 publications, and conducted a synonym merge to improve readability. A density map of the top 50 most frequent keywords was plotted using VOSviewer, as shown in **Figure 8A**. These keywords include “diabetes mellitus,” “tuberculosis,” “risk factors,” “pulmonary tuberculosis,” “prevalence,” “impact,” “Mycobacterium tuberculosis,” “treatment outcomes,” “infection,” “immunity,” “association,” “disease,” “type 2 diabetes,” “expression,” “latent tuberculosis,” “epidemiology” and so on. These keywords represent the focal points of research in the field. CiteSpace was used for clustering of keywords, as shown in **Supplementary Table S5** and **Figure 8B**, which displays eight clusters for visualizing emerging trends in the DM-TB field. The parameters were configured as follows: temporal scope spanning 1995–2023 with 1-year slices, node type defined as keywords, g-index standardization (k=25), and pruning disabled. The cluster network includes 578 nodes and 3,227 links, with Modularity Q = 0.3982 (>0.3), indicating significance and credibility. Weighted Mean Silhouette S = 0.7564 (>0.7) signals homogeneity among clusters, demonstrating a reliable network. The eight clusters are: “multidrug-resistant tuberculosis (MDR-TB),” “murine model,” “cells,” “population pharmacokinetics,” “risk factors,” “T2DM,” “transient hyperglycemia” and “outcomes (show as outcm in figure).”

The top 17 keywords in the DM-TB research burst analysis are shown in **Figure 8C**. Burst strength is calculated by combining keyword frequency with time aspects, where higher strength signifies a sudden or explosive increase in prevalence over a specified period. “Impact” is the most prominent keyword, followed by “manifestations” and “latent tuberculosis.” Notably, the keyword “health” had the longest burst duration. Recently burst keywords in the past three years include “latent tuberculosis,” “inflammation,” and “insulin resistance,” indicating these subjects currently constitute research frontiers in the field. As displayed in **Figure 8D**, the thematic evolution diagram depicting horizontal spatial shifts of nodes constitutes a comprehensive timeline of initial appearances and evolution processes of relevant keywords in academic literature, highlighting shifts in research directions over time and providing insights into the research process. Between 1995 and 2016, research themes focused on hyperglycemia, comorbidities, cytokines, and epidemiology, evolving to tuberculosis, diabetes, as well as latent tuberculosis, immunity, biomarkers, and macrophages from 2017 to 2020. From 2021 to 2023, themes shifted to tuberculosis, latent tuberculosis, HIV, screening, and prediabetes, reflecting the dynamic evolution of DM-TB research.

## 4 Discussion

The incidence of DM-TB has been rising steadily, becoming a focal point in global public health. This study employed bibliometric methods to analyze literature related to DM-TB published between 1995 and 2023, aiming to uncover research hotspots and thematic trends in this field. The analysis encompassed 791 papers published by 1,430 institutions across 100 countries/regions in 263 journals, providing a macro perspective on the current research status and forecasting future trends.

The increasing annual number of publications highlights the robust development. The low research output during 1995–2007 reflects limited academic understanding of the DM-TB association. The marked increase in publications post-2008 may be attributed to studies demonstrating DM elevates TB risk (27), which prompted global enhancements in comorbid disease screening. During the COVID-19 pandemic, shifted research priorities and resource allocation led to reduced DM-TB research output compared to previous periods, accompanied by declining citation rates (39). However, the peak publication volume in 2022 likely reflects concentrated recovery of previously suppressed scientific activities as pandemic management transitioned into routine phases. The United States emerged as a key contributor in terms of publication volume, BC, total link strength, and citation numbers, indicating its strong international influence in this research field. This phenomenon may be attributed to the significant advantages of the U.S. scientific research system. The United States boasts a large number of widely distributed research institutions, and its highly developed economic system provides a solid material foundation for scientific research activities. In contrast, as developing countries and high TB burden countries defined by the WHO, China and India face more complex public health challenges. In recent years, with the growing prominence of DM-TB issues, research investment in the field of DM-TB in these countries has increased significantly. According to data released by the WHO, most high TB burden countries are low- and middle-income nations, where relatively weak healthcare systems, poor nutritional status, and suboptimal living conditions interact, further exacerbating the prevalence of DM-TB (40). Therefore, research on the epidemiology, clinical management, and prevention strategies of DM-TB is particularly critical in these countries.

At the institutional level, the London School of Hygiene & Tropical Medicine and the International Union Against TB and Lung Disease published the highest number of papers on DM-TB research. The London School of Hygiene & Tropical Medicine’s outstanding performance in publication volume, citation frequency, and TLS reflects its leading role and extensive collaborative relationships in this field. At the author level, prolific authors such as Kornfeld, Hardy, Babu, Subash, and Kumar, Nathella Pavan demonstrate marked activity and output, while the significant citation frequency and H-index of Kapur, Anil showcases the major impact of his scientific contributions. These authors form distinct clusters with stable collaborations, providing robust support for research advancements in the field.

In terms of journals, the International Journal of Tuberculosis and Lung Disease and PLOS ONE rank among the top in publication volume and citation frequency, highlighting their authority and influence in the field. Particularly, PLOS ONE’s open-access model offers unrestricted access and citation of research results to scholars worldwide, promoting the widespread dissemination of academic findings. Additionally, the top ten cited publications are all reviews, mainly addressing the epidemiology of DM-TB, the association between DM and TB, and the impact of DM on TB clinical manifestations and treatment outcomes. These areas form the foundation of DM-TB-related research and foster deeper development in the field. Notably, in recent years, themes related to “metformin” have been prominent among co-cited references in the DM-TB research field, becoming a persistent research theme. Studies indicate that metformin may enhance innate immunity against MTB through direct mechanisms or by modulating corticosteroid hormones (41), and its therapeutic use has been associated with reduced mortality rates among patients with DM-TB comorbidity (42).

Through high-frequency keyword extraction and clustering analysis, we identified research hotspots in DM-TB between 1995 and 2023 and predicted future trends in this research field. Clustering analysis indicates that MDR-TB, animal models, cellular and molecular mechanisms, pharmacokinetics, and risk factors are the current research hotspots. Notably, the sudden increase in keywords such as “latent tuberculosis,” “inflammation,” and “insulin resistance” suggests significant potential for future research development in these areas.

Emerging evidence highlights that MDR-TB has evolved into a major public health challenge. Accumulating clinical evidence demonstrates DM serves as an independent risk factor for developing MDR-TB and predicts suboptimal clinical outcomes (43). While advancements in pharmacokinetic modeling have revolutionized tuberculosis drug development, novel therapeutic paradigms focusing on host-pathogen interactions (HPI) are gaining prominence. These host-directed therapies (HDT) represent a promising frontier for optimizing bacterial clearance while preventing post-tuberculosis pulmonary sequelae (44). Similarly, adjunctive therapeutic strategies utilizing natural products have shown potential clinical value. Current evidence confirms that mulberry extracts facilitate glucose absorption, enhance insulin biosynthesis/secretion, and demonstrate antioxidative/anti-inflammatory properties (45). Polyphenolic compounds including quercetin, curcumin, and resveratrol exert therapeutic effects through mitigating intracellular oxidative stress and attenuating chronic low-grade inflammation (46). However, the clinical translation of these interventions faces critical scientific challenges, necessitating implementation of hyperspectral imaging technology for authentication of natural product integrity (47). Thematic trajectory analysis reveals an important paradigm shift in DM-TB research: from initial clinical characterization of disease manifestations to current mechanistic investigations at molecular levels. Notably, the precise pathogenesis of TB in individuals with DM remains poorly delineated. Capitalizing on recent breakthroughs in multi-omics technologies, priority should be given to interdisciplinary research integrating metabolic profiling, immune phenotyping, and transcriptomic signatures to establish predictive biomarkers and therapeutic targets (48). These studies not only enhance our understanding of the pathophysiological mechanisms of DM-TB but also provide a solid theoretical foundation for developing more effective prevention, diagnosis, and treatment strategies.

Despite offering a macro perspective on the research status and future trends within the DM-TB field through bibliometric methods, this study has certain limitations. The research data is solely sourced from the WoSCC database, possibly overlooking relevant studies from other databases. Additionally, the bibliometric tools used may introduce data processing bias. Lastly, the time constraints of the study might lead to the omission of some high-quality articles. Future research should delve deeper into data analysis, extract more valuable information, consider future research directions, and propose concrete suggestions and outlooks. Despite these limitations, the research directions established in this study will contribute to the continuous advancement of the DM-TB field.

## 5 Conclusions

The bibliometric analysis of literature from 1995 to 2023 revealed significant growth in the DM-TB field, with a peak in research activity noted in 2022. The United States and China are major contributors to this research domain, with institutions like the London School of Hygiene & Tropical Medicine standing out. Open-access journals such as PLOS ONE have facilitated the wide dissemination of research findings. Research hotspots focus on areas like MDR-TB, animal models, and pharmacokinetics, indicating future research directions.

While this study highlights the research dynamics and future trends in the DM-TB field, it is limited by data sources and temporal scope. Future studies should aim to deepen data analysis and explore new research directions. These findings are anticipated to further propel the DM-TB field’s development, positively impacting global public health.

## Data Availability

The original contributions presented in the study are included in the article/**Supplementary Material**. Further inquiries can be directed to the corresponding author/s.
