# Supplementary material for: Bibliometric analysis and knowledge mapping of diabetes mellitus combined with tuberculosis research: trends from 1995 to 2023
Source: Front Immunol. 2025 Apr 4;16:1571123. doi: 10.3389/fimmu.2025.1571123 (PMC12006080; doi:10.3389/fimmu.2025.1571123)
Supplement: Supplementary file 1 [file Table1.docx]

**Tabe S1. Ten authors with the most publications**

| **Rank** | **Author** | **Country** | **Documents** | **H-index ^a^** | **G-index ^b^** | **M-index ^c^** | **Citations** | **Year-start ^d^** |
| --- | --- | --- | --- | --- | --- | --- | --- | --- |
| 1 | Kornfeld, Hardy | USA | 30 | 18 | 30 | 1 | 1188 | 2007 |
| 2 | Babu, Subash | India | 27 | 16 | 26 | 1.333 | 720 | 2013 |
| 3 | Kumar, Nathella Pavan | India | 26 | 16 | 24 | 1.333 | 603 | 2013 |
| 4 | Van Crevel, reinout | Netherlands | 26 | 17 | 26 | 0.895 | 1156 | 2006 |
| 5 | Critchley, Julia A | England | 24 | 12 | 24 | 0.75 | 881 | 2009 |
| 6 | Harries, Anthony D | England | 24 | 17 | 24 | 1.063 | 1802 | 2009 |
| 7 | Alisjahbana, Bachti | Indonesia | 21 | 12 | 21 | 0.632 | 1220 | 2006 |
| 8 | Kapur, Anil | Denmark | 21 | 20 | 21 | 1.25 | 1829 | 2009 |
| 9 | Viswanathan, Vijay | India | 21 | 13 | 21 | 1 | 622 | 2012 |
| 10 | Restrepo, Blanca I | USA | 20 | 18 | 20 | 0.947 | 1180 | 2006 |

a. H-index: A measure of scholarly productivity and impact, defined as the maximum value h where an author has h publications each cited at least h times..

b. G-index: A quantitative measure of a researcher’s academic impact, defined as the largest number (g) such that the top g papers, ranked in decreasing order of citations received, have at least g² total citations.

c. M-index: A time-normalized metric assessing the sustainability of a researcher's academic impact.

^d^. Year-start: The year in which an author began publishing papers.
