# Supplementary material for: Bibliometric analysis and knowledge mapping of diabetes mellitus combined with tuberculosis research: trends from 1995 to 2023
Source: Front Immunol. 2025 Apr 4;16:1571123. doi: 10.3389/fimmu.2025.1571123 (PMC12006080; doi:10.3389/fimmu.2025.1571123)
Supplement: Supplementary file 2 [file Table2.docx]

**Table S2. Ten journals with the most publications.**

| **Rank** | **Journal** | **Country** | **Documents** | **Citations** | **TLS** | **IF** | **JCR quantile ranking *** |
| --- | --- | --- | --- | --- | --- | --- | --- |
| 1 | INTERNATIONAL JOURNAL OF TUBERCULOSIS AND LUNG DISEASE | France | 60 | 1464 | 830 | 3.8 | Q2 |
| 2 | PLOS ONE | USA | 60 | 2048 | 712 | 2.9 | Q1 |
| 3 | BMC INFECTIOUS DISEASES | England | 39 | 521 | 366 | 3.4 | Q2 |
| 4 | TUBERCULOSIS | USA | 27 | 446 | 254 | 2.8 | Q3 |
| 5 | TROPICAL MEDICINE & INTERNATIONAL HEALTH | England | 24 | 1028 | 459 | 2.6 | Q2 |
| 6 | INTERNATIONAL JOURNAL OF INFECTIOUS DISEASES | England | 18 | 249 | 187 | 4.8 | Q1 |
| 7 | SCIENTIFIC REPORTS | England | 16 | 318 | 205 | 3.8 | Q1 |
| 8 | CLINICAL INFECTIOUS DISEASES | USA | 15 | 1101 | 385 | 8.2 | Q1 |
| 9 | BMJ OPEN | England | 13 | 135 | 152 | 2.4 | Q1 |
| 10 | FRONTIERS IN IMMUNOLOGY | Switzerland | 11 | 121 | 73 | 5.7 | Q1 |

^*^ JCR quantile ranking: Journals within the same academic field are categorized according to specific standards to intuitively reflect their academic quality and impact.
