# Supplementary material for: Bibliometric analysis and knowledge mapping of diabetes mellitus combined with tuberculosis research: trends from 1995 to 2023
Source: Front Immunol. 2025 Apr 4;16:1571123. doi: 10.3389/fimmu.2025.1571123 (PMC12006080; doi:10.3389/fimmu.2025.1571123)
Supplement: Supplementary file 3 [file Table3.docx]

**Table S3. Ten Cited Journal with the most Ciations.**

| **Rank** | **Cited Journal** | **Country** | **Ciations** | **TLS** | **IF** | **JCR quantile ranking** |
| --- | --- | --- | --- | --- | --- | --- |
| 1 | PLOS ONE | USA | 1574 | 53651 | 2.9 | Q1 |
| 2 | INTERNATIONAL JOURNAL OF TUBERCULOSIS AND LUNG DISEASE | France | 1379 | 42447 | 3.8 | Q2 |
| 3 | CLINICAL INFECTIOUS DISEASES | USA | 824 | 27900 | 8.2 | Q1 |
| 4 | DIABETES CARE | USA | 692 | 22296 | 14.8 | Q1 |
| 5 | TROPICAL MEDICINE & INTERNATIONAL HEALTH | England | 630 | 22076 | 2.6 | Q2 |
| 6 | PLOS MEDICINE | USA | 595 | 18211 | 10.5 | Q1 |
| 7 | LANCET | England | 446 | 14244 | 98.4 | Q1 |
| 8 | JOURNAL OF IMMUNOLOGY | USA | 372 | 16181 | 3.6 | Q2 |
| 9 | TUBERCULOSIS | England | 363 | 14273 | 2.8 | Q3 |
| 10 | JOURNAL OF INFECTIOUS DISEASES | USA | 359 | 14158 | 5 | Q1 |
