# Supplementary material for: Bibliometric analysis and knowledge mapping of diabetes mellitus combined with tuberculosis research: trends from 1995 to 2023
Source: Front Immunol. 2025 Apr 4;16:1571123. doi: 10.3389/fimmu.2025.1571123 (PMC12006080; doi:10.3389/fimmu.2025.1571123)
Supplement: Supplementary file 4 [file Table4.docx]

**Table S4. Ten highly cited references.**

| **Rank** | **Title** | **Author** | **Year** | **Journal** | **TLS** | **Citations** |
| --- | --- | --- | --- | --- | --- | --- |
| 1 | Diabetes mellitus increases the risk of active tuberculosis: a systematic review of 13 observational studies | Jeon CY | 2008 | PLOS MEDICINE | 4799 | 416 |
| 2 | The impact of diabetes on tuberculosis treatment outcomes: a systematic review | Baker MA | 2011 | BMC MEDICINE | 3402 | 272 |
| 3 | Tuberculosis and diabetes mellitus: convergence of two epidemics | Dooley KE | 2009 | LANCET INFECTIOUS DISEASES | 2394 | 211 |
| 4 | The effect of type 2 diabetes mellitus on the presentation and treatment response of pulmonary tuberculosis | Alisjahbana B | 2007 | CLINICAL INFECTIOUS DISEASES | 1932 | 126 |
| 5 | Association of diabetes and tuberculosis: impact on treatment and post-treatment outcomes | Jiménez-corona ME | 2013 | THORAX | 1773 | 117 |
| 6 | Diabetic control and risk of tuberculosis: a cohort study | Leung CC | 2008 | AMERICAN JOURNAL OF EPIDEMIOLOGY | 1682 | 111 |
| 7 | Tuberculosis and diabetes in southern Mexico | Ponce-de-leon A | 2004 | DIABETES CARE | 1455 | 100 |
| 8 | Diabetes and tuberculosis: the impact of the diabetes epidemic on tuberculosis incidence | Stevenson CR | 2007 | BMC PUBLIC HEALTH | 1428 | 98 |
| 9 | Impact of Diabetes Mellitus on Treatment Outcomes of Patients with Active Tuberculosis | Dooley KE | 2009 | AMERICAN JOURNAL OF TROPICAL MEDICINE AND HYGIENE | 1372 | 92 |
| 10 | Bi-directional screening for tuberculosis and diabetes: a systematic review | Jeon CY | 2010 | TROPICAL MEDICINE & INTERNATIONAL HEALTH | 1396 | 91 |
