# Supplementary material for: Bibliometric analysis and knowledge mapping of diabetes mellitus combined with tuberculosis research: trends from 1995 to 2023
Source: Front Immunol. 2025 Apr 4;16:1571123. doi: 10.3389/fimmu.2025.1571123 (PMC12006080; doi:10.3389/fimmu.2025.1571123)
Supplement: Supplementary file 5 [file Table5.docx]

**Table S5. Eight Keywords cluster.**

| **Cluster** | **Size ^a^** | **Silhouette ^b^** | **Mean (Year)** | **Label** | **Other Keywords** |
| --- | --- | --- | --- | --- | --- |
| 0 | 90 | 0.717 | 2013 | multidrug-resistant tuberculosis | treatment outcome; treatment outcomes; public health; integration |
| 1 | 78 | 0.655 | 2017 | murine model | type 2 diabetes mellitus; mechanisms; diabetes mellitus; in vivo |
| 2 | 75 | 0.744 | 2014 | cells | vitamin d; expression; biomarkers; b cells |
| 3 | 62 | 0.746 | 2013 | population pharmacokinetics | plasma concentrations; rifampin; pharmacokinetics; multidrug resistant tuberculosis |
| 4 | 60 | 0.748 | 2011 | risk factors | India; body mass index; burden; prevalence |
| 5 | 55 | 0.715 | 2015 | t2dm | single nucleotide polymorphisms; bias; serum 25(oh)d; interaction |
| 6 | 54 | 0.846 | 2005 | diabetes mellitus | transient hyperglycemia; stress-induced hyperglycemia; transcriptome; IFN-gamma |
| 7 | 33 | 0.833 | 2017 | diabetes complications | outcome; glycemic control; trends; disease |

^a^. Size: Usually refers to the size of a node, which represents the importance or frequency of a keyword within a cluster. A larger size indicates higher frequency or greater importance of the keyword in the cluster.

^b^. Silhouette: The S value is typically used to assess the validity and reliability of clustering. Generally, if S > 0.5, the clustering is considered reasonable. If S > 0.7, the clustering is deemed convincing, with a high level of consistency among keywords within the cluster.
