# Supplementary material for: Bibliometric analysis and knowledge mapping of diabetes mellitus combined with tuberculosis research: trends from 1995 to 2023
Source: Front Immunol. 2025 Apr 4;16:1571123. doi: 10.3389/fimmu.2025.1571123 (PMC12006080; doi:10.3389/fimmu.2025.1571123)
Supplement: Supplementary file 6 [file DataSheet1.docx]

TS=("Diabetes Mellitus" OR "diabetes" OR "diabetic mellitus" OR "diabetic" OR "Diabetes Mellitus,Type 2" OR "Diabetes Mellitus,Type 1" OR "Type 2 Diabetes Mellitus" OR "Type 1 Diabetes Mellitus" OR "Type 2 Diabetes" OR "Type 1 Diabetes" OR "Diabetes, Type 2" OR "Diabetes, Type 1" OR "Diabetes Mellitus, Non Insulin Dependent" OR "Noninsulin-Dependent Diabetes Mellitus" OR "Diabetes Mellitus, Type II" OR "Stable Diabetes Mellitus”OR “Diabetes Mellitus, Insulin Dependent" OR "Insulin-Dependent Diabetes Mellitus" OR "Diabetes Mellitus, Type I" OR "NIDDM" OR "IDDM" OR "DM") AND TS=( "Mycobacterium Tuberculosis" OR "Tuberculosis" OR "Pulmonary Tuberculosis" OR "Tuberculoses" OR "Kochs Disease" OR "Koch's Disease" OR "Koch Disease" OR "PTB" OR "TB")
